# Relationship between antimicrobial resistance and virulence factors in uropathogenic *Escherichia coli* isolates from Ramadi, Iraq: phenotype and genotype identification

**DOI:** 10.4314/ahs.v23i3.56

**Published:** 2023-09

**Authors:** Ahmed Dhahir Abed, Thamer Y Mutter

**Affiliations:** Department of Biology, College of Science, University of Anabr, Ramadi, Anbar, Iraq

**Keywords:** UPEC resistance profile, virulence factor, Biofilm formation, hemagglutinin, hemolysin

## Abstract

**Background:**

Uropathogenic *Escherichia coli* (UPEC) is the most predominant pathogen that causes severe urinary tract infections (UTIs). Their therapeutic options are limited due to the rising of antibiotic resistance.

**Objective:**

The aim of the study was to evaluate the level of antibiotic resistance profile, redundancy of virulence genes, and their correlation.

**Methods:**

41 UPEC isolates were collected from patients diagnosed with UTI, identified by the standard microbiological analysis, and tested for susceptibility to 12 antibiotic agents using the Kirby-Bauer method. The ability of UPEC isolates to produce biofilm, hemolyze and cause clumping of blood was determined. Virulence genes were detected by PCR analysis.

**Results:**

The percentage of UPEC isolates was higher in females (78.1%) than in males (21.9%). UPEC isolates showed a high degree of resistance towards Ceftriaxone (90.2%), Cefepime (90.2%), Ciprofloxacin (82.9%), Levofloxacin (82.9%), and Trimethoprim-Sulfamethoxazole (80.4%). Biofilm formation (87.8%) and hemagglutinin activity (80.4%) were the most predominant virulence markers expressed in UPEC and showed a high degree of correlation with the antibiotic resistance profile. PCR analysis showed that *fimH* (85.3%) was the most prevalent gene detected in UPEC isolates, followed by *aac3-II* (80.4%) among the five genes tested, *bla_TEM_*, *aac3-II, sul2, hlyA*, and *fimH*. The correlation between antibiotic resistant patterns and the presence *aac3*-II gene was significantly high. The resistance to the sulfonamides' combined antibiotic was highly correlated with the presence of *sulf2* gene.

**Conclusion:**

Antimicrobial resistance was significantly linked to phenotypic and genotypic virulence factors. These results will aid in elucidating the pathogenicity of UTIs and guiding treatment decisions.

## Introduction

One of the most prevalent outpatient bacterial illnesses is urinary tract infection (UTI), which affects more than 50 % of all adults at some point in their lives^1^. The urethra and bladder are impacted by lower UTIs. On the other hand, upper UTIs damage kidney function and can even be fatal when bacteria from infected kidneys enter the bloodstream^2^.

*Escherichia coli (E. coli)* is still the most commonly found uropathogen. Uropathogenic *E. coli* (UPEC) was isolated as the most common infectious pathogen associated with both complicated and uncomplicated UTIs^3^. UPEC are strains that are able to infect and cause serious illness in the urinary system. These strains are genetically differentiated from the normal flora of the gastrointestinal system^4^.

*E. coli* is a microorganism's normal flora inhabiting the human gut (commensal microorganisms). Some of these strains can become more pathogenic than their commensal counterparts. In order to adapt to new environments and increase the bacterial capacity to cause a wide range of illnesses, these strains acquire specialized virulence factors through horizontal gene transfer mediated by transposons, plasmids, bacteriophages, and pathogenicity islands 5. They can present in the gut without causing any harm; however, they have the potential to spread and colonize other host niches, such as the urinary tract, the blood, and the central nervous system leading to several diseases^6^. UPEC pathogenicity is associated with the presence of several virulence factors such as antibiotic resistance genes, toxins, adhesins, and siderophores chelating agents that facilitate resistance to multi antibiotics, damage host surfaces, and capture iron which in turn avoids the host defense system^7^.

The virulence of *E. coli* is the consequence of the interaction of multiple unique virulence factors, which serve to distinguish potentially pathogenic strains from non-pathogenic strains^8^. *E. coli* pathogenicity during a specific infection depends on the existence and actual expression of the virulence genes within them and on the host's environmental factors^9^.

Globally, the rate of *E. coli* strains is increasing in developing resistance to most antibiotics prescribed for UTIs treatment. It is crucial to be aware of regional UPEC susceptibility so the right prescription of antibiotics can be used to treat UTIs caused by *E. coli*^10^.

Resistance to antibiotics, the capacity of adherence (fimbriae that help in adhesion and tissue invasion), synthesis of hemolysin (secreted virulence factor that helps in colonizing the urinary tract), and biofilm formation are all the major determinant factors that specify UPEC^11^.

Recently, most attention is paid to blaTEM genes that are carried on the plasmid and one of the main problems that cause pathogens to resist newer generations beta-lactam antibiotics^12^,^13^,^14^. *sul2* is the most common among enterobacteria that are associated with sulfonamides resistant. Also, *aac(3)-II* aminoglycosides resistance is highly associated with *E. coli* resistance that carried on the plasmid^14^.

*fimH* and *hlyA* are the most associated virulence factors with *E. coli* pathogenicity. These virulence factors are necessary for the bacteria to invade and colonize the urinary system and remain in spite of the well-functioning host defensive mechanisms^15^.

Increasing the resistance of UPEC strains to multi antibiotics elevated a serious threat to global health^16^. Therefore, it is important for periodic screening of the local prevalence of UPEC strains to control their antibiotic resistance profile. Moreover, monitoring the distribution of virulence gene markers associated with antibiotic resistance can keep our community from infecting with strains have high resistance or acquired new resistance to antibiotics. For that, the aim of this study was to evaluate the distribution of antibiotic resistance among UPEC associated with virulence markers in UTI patients in Ramadi region, Iraq.

## Materials and methods

### Sample Collection and Identification of uropathogens

*E. coli* was isolated from 120 patients suffering from UTI from December 2021 to March 2022. At the same time, 20 commensal E. coli strains were isolated from feces of healthy individuals that never had UTIs as a control. Also, a well-designed questionnaire was used to collect clinical data from patients and control (the questionnaire form is presented in the Supplementary Appendix, table S1).

Urine samples were collected from UTI patients, mid-stream urine, using a sterile container provided for this purpose. In addition to the symptomatic for UTI, a concentration of > 105 colony forming unit (CFU) for every single cultured organism was considered UTI positive^17^. Urine samples were processed directly, within 30 minutes, after sample collection in the microbiology lab in each hospital (AlRamadi Teaching Hospital for Maternity and children and Ramadi Teaching Hospital). UPEC strains were identified using cultural media such as MacConkey agar and Eosin Methylene blue agar, and all isolates were confirmed by conventional biochemical tests^18^. In addition, VITEK-2 compact system (Biomérieux, France) was used for further confirmation of UPEC strains.

### Biofilm formation

Congo red agar method was used to detect biofilm formation in UPEC and control. The medium was prepared by mixing 37 gm/L brain heart broth, 50 gm/L sucrose, 0.8 gm/L Congo red stain (prepared separately), and 10 gm/L agar-agar. The isolates were cultured on the medium and incubated aerobically at 37 °C for 24 h. *E. coli* isolates that produced black coloured colonies with a dry crystalline consistency were considered biofilm producers 19.

### Hemolysin activity

UPEC and control isolates were inoculated into peptone water and incubated at 37 °C until the turbidity of the growth reached 0.5 by matching with 0.5 McFarland standard. Blood base agar supplemented with 5% sheep blood was sub cultured with the organisms from peptone water growth and incubated at 37 °C for overnight. Hemolysin activity was observed through complete hemolysis or Beta hemolysis on the blood agar plates that indicated by a clear zone around the colonies^16^.

### Hemagglutination test

The direct bacterial hemagglutination test slide method was used as described by^20^. Isolates were inoculated into 1% freshly made nutrient broth and incubated at 37 °C till full fimbriation. Then, the growth was centrifuged and the sticky colonies, bacillary, resuspended in a small amount of residual broth. One drop of 3% erythrocyte suspension, it was prepared from Human blood (group O) by centrifugation and the Red Blood Cells (RBCs) were washed three times in normal saline and resuspended to a final volume of 3% erythrocyte suspension, was mixed with one drop of the sticky culture on a slide. The slide was kept at room temperature for 10 minutes. If clumping was observed, hemagglutination positive.

### Antimicrobial susceptibility test

All UPEC and control strains were tested for their susceptibility against 12 antimicrobial agents. Antimicrobial susceptibility was assessed by the Kirby-Bauer disk diffusion method and the resistance to the below antibiotics was determined according to the guidelines proposed by the Clinical and Laboratory Standards Institute (CLSI)21. Antimicrobial disks used were Ceftriaxone (COR 10 µg), Ceftazidime (CAZ 30 µg), Cefotaxime (CTX 30 µg), Piperacillin (PI 100 µg), Cefepime (CPM 30 µg), Cefoxitin (CX 30 µg), Amikacin (AK 30 µg), Gentamicin (GM 10 µg), Trimethoprim-sulfamethoxazole (TS 25 µg), Ciprofloxacin (CIP 5 µg), Levofloxacin (LEV 5 µg), and Imipenem (IMP 10 µg). *E. coli* ATCC®25,922TM was used as standard control.

### PCR detection of UPEC virulence factor

Total genomic DNA from UPEC isolates was extracted using the Promega Genomic DNA Purification Kit (USA) by following the manufacturers' instructions. PCR assays were used to detect *blaTEM, aac3-II, sul2, hlyA*, and *fimH* by using the primers listed in Table 1.

**Table 1:** Primers used in the study

Primer	Sequence (5′-3′)	Tm °C	Product length (bp)	Reference
blaTEM-F	ATAAAATTCTTGAAGAC	50	1181	(22)
blaTEM-R	TTACCAATGCTTAATCA			
aac(3)-II-R	TGAAACGCTGACGGAGCCTC	55	369	(23)
aac(3)-II-F				
aac(3)-II-R	GTCGAACAGGTAGCACTGAG			
Sul2-F	CGGCATCGTCAACATAACCT	55	721	(24)
Sul2-R	TGTGCGGATGAAGTCAGCTC			
hlyA-F	AACAAGGATAAGCACTGTTCTGGCT	64	1177	(25)
hlyA-R	ACCATATAAGCGGTCATTCCCGTCA			
FimH-F	GAGAAGAGGTTTGATTTAACTTATTG	60	559	(26)
FimH-R	AGAGCCGCTGTAGAACTGAGG			

Tm: Annealing temperature for specified primer

The amplification reactions were prepared by using Hot PCR Master Mix (Promega, USA). The reaction was carried out in a final volume of 20 µl containing, 10 µl Hot PCR master mix, 10 pmol of each primer, 50 ng of isolated DNA, and the final volume was adjusted to 20 µl using ddH2O. Individually adjusted annealing temperature for DNA amplification conditions were performed for each gene (Table 1). After PCR amplification, 1.5 % of gel agarose was prepared to analyse PCR products. The gel documentation system was used to visualize the PCR product.

### Statistical analysis

Chi-square or Fisher's exact test (two-tailed) were used for analysing and comparing all variables. All statistical analyses were performed using GraphPad Prism version 8, GraphPad Software, San Diego, California USA. P < 0.05 was considered statistically significant.

## Results

During the period from December, 2021 to March, 2022, a total of 41 UPEC isolates were isolated from 120 patients suffering from UTI and 20 isolates were isolated from feces of healthy individuals as control and subjected to this study.

The age of the patients ranged from 18 to 78 years. The majority of the UPEC isolates were isolated from females 32 (78.1%) compared to 9 (21.9%) from males. Biofilm formation was detected in 36 (87.8%) of UPEC compared to 4 (20%) in control isolates, which is statistically highly significant with p value < 0.001. Similarly, 33 (80.4%) of UPEC isolates were hemagglutinin positive while only 4 (20%) isolates showed hemagglutinin positive in control, which is statistically highly significant (p< 0.001). Unlike, the hemolysin test showed no statistical significance between UPEC and control isolates. Out of 41 UPEC isolates, only 9 (21.9%) were hemolysin positive and 2 (10%) in control isolates (Table 2).

**Table 2:** Expression of virulence factors of UPEC compared to control

Virulence Factors	UPEC (n=41)	Control (n=20)	P Value
Biofilm formation	36 (87.8%)	4 (20%)	< 0.001 (significant)
Beta-Hemolysis	9 (21.9%)	2 (10%)	0.312 (Not significant)
Hemagglutinin	33 (80.4%)	4 (20%)	< 0.001 (significant)

*P* value less than 0.05 is statistically significant

UPEC isolates showed a higher degree of resistance to antibiotics compared to control (Table 3). The resistance pattern was similar in UPEC isolates against Ceftriaxone, Cefepime, Piperacillin, and Ceftazidime in 37 (90.2%) compared to control 10 (50%), 7 (35%), 6 (30%), and 8 (40%), respectively (P< 0.001). While the resistance pattern against Amikacin and Gentamicin were 43.9% and 68.2% in UPEC isolates compared to 10% and 20% in control isolates, respectively (P< 0.001). Trimethoprim-Sulfamethoxazole showed resistance to 80.4 % of UPEC compared to 20% of control (P< 0.001). Ciprofloxacin and Levofloxacin resistance were observed in 82.9% of UPEC isolates, while the resistance was observed in 10% for Ciprofloxacin and 15 % for Levofloxacin in control isolates with P value < 0.001. With imipenem, UPEC isolates had the lowest resistance with only 7.3%.

**Table 3:** Antibiotic resistance profile of UPEC compared to control

Antibiotic	UPEC (n=41)	Control (n=20)	P Value
Ceftriaxone	37 (90.2%)	10 (50%)	< 0.001 (significant)
Cefepime	37 (90.2%)	7 (35%)	< 0.001 (significant)
Ceftazidime	37 (90.2)	8 (40%)	< 0.001 (significant)
Cefoxitin	26 (63.4%)	6 (30%)	0.027 (significant)
Cefotaxime	36 (87.8%)	6 (30%)	< 0.001 (significant)
Piperacillin	37 (90.2%)	6 (30%)	< 0.001 (significant)
Amikacin	18 (43.9%)	2 (10%)	0.009 (significant)
Gentamicin	28 (68.2%)	4 (20%)	< 0.001 (significant)
Trimethoprim/Sulfamethoxazole	33 (80.4 %)	4 (20%)	< 0.001 (significant)
Ciprofloxacin	34 (82.9%)	2 (10%)	< 0.001 (significant)
Levofloxacin	34 (82.9%)	3 (15%)	< 0.001 (significant)
Imipenem	3 (7.3%)	2 (10%)	> 0.999 (Not significant)
**MDR**	**36 (87.8%)**	**2 (10%)**	**< 0.001 (significant)**

MDR Multi Drug Resistance; *P* value less than 0.05 is statistically significant

Additionally, more than 87.8% of UPEC isolates were resistant to more than three classes of antibiotics used in this study compared to only 10% in the control which is statistically highly significant (P< 0.001). These are classified as Multi Drug Resistance (MDR).

We then studied the association between the antimicrobial resistance profile and the expression of virulence genes (Table 4). The results showed a high correlation between biofilm formation and the resistance to most antibiotics used in this study. The biofilm producers showed a higher level of resistance. The degree of resistance to COR (97.2% VS 40%, P=0.0036), CPM (97.2% VS 40%, P=0.0036), CAZ (97.2% VS 40%, P=0.0036), CTX (94.4% VS 40%, P=0.0086), PI (97.2% VS 40%, P=0.0036), GM (75% VS 20%, P=0.0284), TS (88.8% VS 20%, P=0.0032), CIP (88.8% VS 40%, P=0.0278), and LEV (88.8% VS 40%, P=0.0278) was significantly higher in biofilm producers compared to non-producers. Similarly, the same resistance pattern was observed in hemagglutinin positive isolates compared to hemagglutinin negative strains. However, the results showed no significant association between antibiotic resistance and hemolysin positivity.

**Table 4:** Relationship between antibacterial resistance in UPEC and expressed virulence factor

Virulence factor	Antibiotic resistance: n (%)											
	
	COR	CPM	CAZ	CX	CTX	PI	AK	GM	TS	CIP	LEV	IMP
Biofilm												
Positive =36	35(97.2%)	35(97.2%)	35(97.2%)	25(69.4%)	34(94.4%)	35(97.2%)	18(50%)	27(75%)	32(88.8%)	32(88.8%)	32(88.8%)	2(5.5%)
Negative=5	2(40%)	2(40%)	2(40%)	1(20%)	2(40%)	2(40%)	0	1(20%)	1(20%)	2(40%)	2(40%)	1(20%)
	
P value	0.0036	0.0036	0.0036	0.0514	0.0086	0.0036	0.0563	0.0284	0.0032	0.0278	0.0278	0.3302
	
Hemolysis												
Positive =9	9(100%)	9(100%)	9(100%)	6(66.6%)	9(100%)	9(100%)	4(44.4%)	6(66.6%)	8(88.8)	9(100%)	9(100%)	0
Negative=32	28(87.5%)	28(87.5%)	28(87.5%)	20(62.5%)	27(84.3%)	28(87.5%)	14(43.7%)	22(68.7%)	25(78.1%)	25(78.1%)	25(78.1%)	3(9.3%)
	
P value	0.5592	0.5592	0.5592	>0.9999	0.5681	0.5592	>0.9999	>0.9999	0.6586	0.3148	0.3148	>0.9999
	
Hemagglutinin												
Positive =33	33(100%)	33(100%)	33(100%)	26(78.7%)	31(93.9%)	33(100%)	15(45.4%)	25(75.7%)	29(87.8%)	31(93.9%)	31(93.9%)	3(9%)
Negative=8	4(50%)	4(50%)	4(50%)	0	4(50%)	4(50%)	3(37.5%)	3(37.5%)	4(50%)	3(37.5%)	3(37.5%)	0
	
P value	0.0007	0.0007	0.0007	<0.0001	0.0086	0.0007	>0.9999	0.0840	0.0333	0.0014	0.0014	>0.9999

P value less than 0.05 is statistically significant

### Detection of virulence and antimicrobial resistance genes by PCR

**Figure 1:**
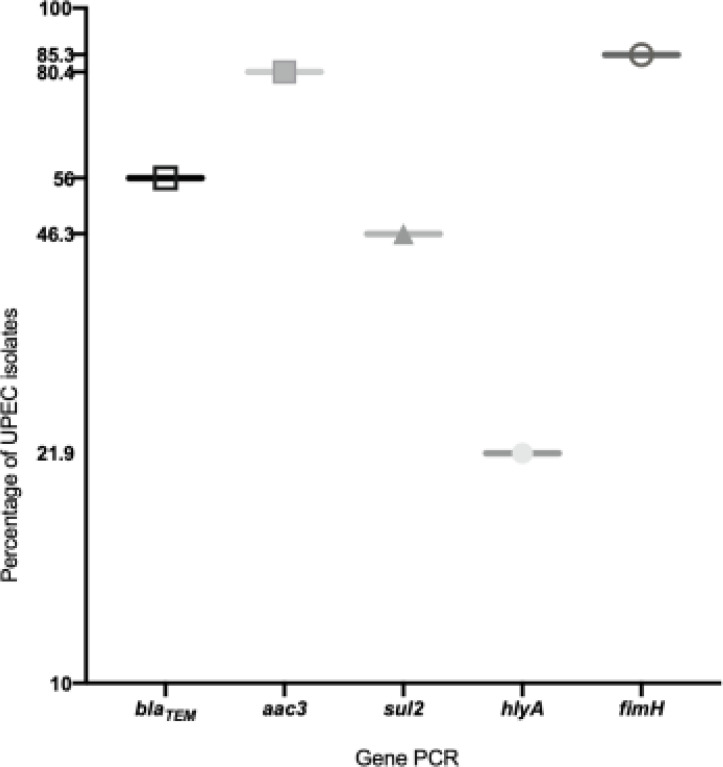
Percentages of Antibiotic resistance genes and virulence genes among UPEC isolates: empty circle refers to the number of UPEC having *fimH;* filled squares refers to *aac3-II*; empty square refers to *bla_TEM_*; triangle refers to *sul2*; filled circle refers to *hlyA*

Two virulence genes (*fimH* and *hlyA*) and three antimicrobial resistance genes (*bla_TEM_, aac3-II*, and *sul2*) were successfully amplified by PCR. Figure 1 shows the number of isolates that carry the tested genes. *fimH* was the most prevalent gene in UPEC (85.3%) followed by *aac3-II* (80.4%), *bla_TEM_* (56%), *sul2* (46.3%), and *hlyA* (21.9%) was the least prevalent gene. The results showed that at least one of the mentioned genes is carried by all of the isolates.

Also, the results showed a high degree of correlation between the *aac3-II* and antimicrobial resistance (Table 5). The isolates carry *aac3-II* were resistance to COR (100%, *P*=0.0007), CPM (100%, *P*=0.0007), CAZ (100%, *P*=0.0007), PI (100%, P=0.0007), CTX (96.9%, *P*=0.0032), CIP (93.9%, *P*=0.0014), LEV (93.9%, *P*=0.0014), TS (87.8%, *P*=0.0333), and GM (81.8%, *P*=0.0005). A high degree of correlation was observed between *sul2* and the two antibiotics, TS (100%, *P*=0.0041) and LEV (100%, *P*=0.0098). Moreover, *fimH* correlation was significantly high with the resistance to AK (51.4%, P=0.0266). There was a statistically high significance between the presence of *sul2* and the resistance to the sulfonamide antibiotic TS (*P*=0.0041). However, the correlation between *blaTEM* and *hlyA* was insignificant with the antibacterial resistance pattern.

**Table 5:** Relationship between antimicrobial resistance in UPEC and PCR virulence factor genes

PCR gene	Antibiotic resistance (n)											
	
	COR	CPM	CAZ	CX	CTX	PI	AK	GM	TS	CIP	LEV	IMP
*bla_TEM_*												
Positive =23	21(91.3%)	21(91.3%)	21(91.3%)	15(65.2%)	21(91.3%)	21(91.3%)	9(39.1%)	16(69.5%)	18(78.2%)	21(91.3%)	21(91.3%)	0
Negative=18	16(88.8%)	16(88.8%)	16(88.8%)	11(61.1%)	15(83.3%)	16(88.8%)	9(50%)	12(66.6%)	15(83.3%)	13(72.2%)	13(72.2%)	3(16.6%)
	
P value	>0.9999	>0.9999	>0.9999	>0.9999	0.6384	>0.9999	0.5394	>0.9999	>0.9999	0.2086	0.2086	0.0765
	
*aac3*												
Positive =33	33(100%)	33(100%)	33(100%)	23(69.6%)	32(96.9%)	33(100%)	16(48.4%)	27(81.8%)	29(87.8%)	31(93.9%)	31(93.9%)	2(6%)
Negative=8	4(50%)	4(50%)	4(50%)	3(37.5%)	4(50%)	4(50%)	2(25%)	1(12.5%)	4(50%)	3(37.5%)	3(37.5%)	1(12.5%)
	
P value	0.0007	0.0007	0.0007	0.1169	0.0032	0.0007	0.4290	0.0005	0.0333	0.0014	0.0014	0.4882
	
*sul2*												
Positive =19	19(100%)	19(100%)	19(100%)	13(68.4%)	19(100%)	19(100%)	10(52.6%)	15(78.9%)	19(100%)	18(94.7%)	19(100%)	0
Negative=22	18(81.8%)	18(81.8%)	18(81.8%)	13(59%)	17(77.2%)	18(81.8%)	8(36.3%)	13(59%)	14(63.6%)	16(72.7%)	15(68.1%)	3(13.6%)
	
P value	0.1105	0.1105	0.1105	0.7460	0.0507	0.1105	0.3550	0.2000	0.0041	0.0994	0.0098	0.2354
	
*hlyA*												
Positive =9	9(100%)	9(100%)	9(100%)	6(66.6%)	9(100%)	9(100%)	4(44.4%)	6(66.6%)	8(88.8%)	9(100%)	9(100%)	0
Negative=32 28(87.5%)		28(87.5%)	28(87.5%)	20(62.5%)	27(84.3%)	28(87.5%)	14(43.7%)	22(68.7%)	25(78.1%)	25(78.1%)	25(78.1%)	3(9.3%)
	
P value	0.5592	0.5592	0.5592	>0.9999	0.5681	0.5592	>0.9999	>0.9999	0.6586	0.3148	0.3148	>0.9999
	
*fimH*												
Positive =35	32(91.4%)	32(91.4%)	32(91.4%)	22(62.8%)	32(91.4%)	32(91.4%)	18(51.4%)	24(68.5%)	29(82.8%)	29(82.8%)	29(82.8%)	2(5.7%)
Negative=6	5(83.3%)	5(83.3%)	5(83.3%)	4(66.6%)	4(66.6%)	5(83.3%)	0	4(66.6%)	4(66.6%)	5(83.3%)	5(83.3%)	1(16.6%)
	
P value	0.4830	0.4830	0.4830	>0.9999	0.1476	0.4830	0.0266	>0.9999	0.5777	>0.9999	>0.9999	0.3860

*P* value less than 0.05 is statistically significant

## Discussion

*E. coli* is the most prevalent infectious pathogen that causes UTIs and affects people at least once in their lifetime. UTI has become a major threat to public health due to the high increase in antibiotic resistance. The antibiotic resistance pattern of UPEC isolates and the association with phenotypic and genotypic characteristics in patients diagnosed with UTI are not well known, especially in Iraq. Few studies worldwide studied some of these characteristics and their association with antibiotic resistance^14^,^15^,^16^,^27^,^29^. Our results showed that UTIs were most prevalent in females. The percentage is in agreement with previous studies^15^,^30^.

UTI is more common in females due to structural differences. *E. coli* is one of the microbes that inhabit the large intestine as normal flora, and faecal contamination is very common in the urinary tract in females due to the fact that the urethra is wider and shorter that makes it less effective in preventing the intestinal flora from getting through the urinary tract^30^,^31^.

Antibiotic resistance testing results of isolated *E. coli* showed a high degree of resistance to most antibiotics used in this study. UPEC isolates were highly resistant to Beta-lactamase antibiotics, followed by quinolones, sulfonamides, aminoglycosides, and carbapenems. This indicates that UPEC isolates have a high degree of resistance narrowing the choices to use the proper medication. The high percentage of resistance could be self-medication, as in our region, there are no strict roles for prescriptions and pharmacists, and even patients, can prescribe drugs and consume it without medicinal prescription. Also, the high percentage of genetic transfer between isolates increased the resistance to antibiotics^32^.

However, the lowest resistance rate among UPEC isolates was against IMP (7.3%) and AK (43.9%). The results agree with previous studies that showed UPEC isolates were more sensitive to IMP and AK than other antibiotics^33^,^34^.

Our results indicated that the percentage of MDR among UPEC was higher compared to other studies in North America and Europe 35, 36. However, the percentage of MDR isolates agrees with most studies done in developing countries^37^. Inappropriate use of antibiotics (excessive use of broad-spectrum antibiotics), self-medication, and most importantly, inadequate prescriptions of antibiotics without antibiotic susceptibility testing are the main reasons for spreading of MDR UPEC in developing countries^38^. So, it is necessary to check the local resistance profile of UPEC to determine the best choice for UTI treatment.

Several virulence factors aided UPEC in invading and colonizing host cells^39^. The level of pathogenicity in UPEC strains is linked to the expression of these virulence factors^11^. Determination of these virulence factors and their correlation with antibiotic resistance in UPEC could help to identify the level of pathogenesis of UTIs and prevent its consequences, such as complicated UTIs and renal failure^28^,^40^. We examined the ability of isolated UPEC to produce biofilm, hemagglutinin, and hemolysin activity. Biofilm formation is one of the most virulent factors found in pathogenic bacteria. Biofilm helps protect UPEC from host immunity and raises bacteria's persistence by increasing the resistance to antibacterial agents. Our results indicated higher biofilm formation in UPEC with 87.8% compared to 20% in control. This result agrees with several studies that indicated that biofilm was the prevalent virulence factor among UPEC isolates^15^,^16^,^27^,^31^,^40^.

This reflects the high resistance to the 3rd generation of cephalosporins, sulfonamides, and quinolones classes used in this study. Biofilm structure decreases the concentration of antibiotic agents and delays the penetration into the bacterial cells which causes insufficient concentration of antibiotics^41^. Also, hemagglutinin production showed a high degree of correlation with the resistance of UPEC towards most of the antibiotics used in this study. The expression of hemagglutinin plays a major role in facilitating the adhesion of isolates to the surface of uroepithelium and then biofilm formations and colonization of the host^42^. This is in accordance with the high number of biofilm formation in UPEC isolated in our study.

The study showed that 21.9% of UPEC were hemolysin positive compared to 10% in control. Even though all hemolysin positive UPEC were resistant (100%) to most of the antibiotics used in this study, there was no significant correlation between hemolysin production and antibiotic resistance pattern. This result is consistent with other studies done in Iraq and neighbouring countries^34^,^37^. Hemolysin activity is more frequent in pyelonephritis, suggesting that this virulence factor may play a significant role in the pathogenesis of UPEC in pyelonephritis patients^43^.

In this study, five genes were detected by PCR amplification. We wanted to study the correlation between the genotypic and phenotypic resistance among UPEC isolated in this study.

The *aac3-II* showed a high degree of correlation with aminoglycosides resistance, specifically with GM (P=0.0005). Similarly, *sul2* was correlated with sulfonamides resistance (TS) (P=0.0041).

Even though *fimH* was the most prevalent among UPEC isolates, the results showed no correlation between the mentioned gene and the resistance pattern. *fimH* encoded type 1 fimbriae that helps in adherence and biofilm formation in UTI of animal models and their function is not really known in human pathology. Several studies on the murine UTIs model showed that type 1 fimbriae activate mucosal inflammation, increases bacterial survival, and helps in biofilm formation^44^. However, the function of the type 1 fimbriae in human UTI remains controversial due to their expression in both pathogenic and commensal strains^45^.

The correlation between the presence of *bla_TEM_* and antibiotic resistance was not significant which could be to the fact that *bla_TEM_* genes encoded broad spectrum enzymes that are responsible for the resistance to amino-pencillins^14^. Bacteria can create a variety of drug-resistance mechanisms so that specific genes cannot correlate with phenotypic antibiotic resistance, such as mutations that lead to structural alterations at the site of the drug's action or a shift in the antibiotic's action point^46^. Furthermore, the efflux pump system is widely known in *E. coli* phenotypic resistance to beta-lactam antibiotics^47^. The result is consistence with a study by Adamus-Białek et al (2018), they showed that even though *bla_TEM_* is present in almost all UPEC in their study, there was no correlation between the mentioned gene and the resistance profile^14^. Our results showed that there is a significant and not significant correlation between antibiotic resistance profile and virulence factors (expressed gene or PCR analysis). The correlation between the two variables in UPEC is not always significant or related and it depends on sample size, type of *E. coli* isolated and type of the illness, resistance profile that could differ according to the region, and the type of antibiotic used in the study^48^. Moreover, gradual evolutionary mechanisms responsible for antibiotic resistance may occur at any time due to continual exposure to drugs^49^.

## Conclusion

Our study showed high associations between antibiotic resistance profile and both phenotypic and genotypic virulence factors. Our results indicated that Biofilm formation and hemagglutinin activity were the most predominant virulence markers expressed in UPEC and showed a high degree of correlation with antibiotic resistance profile. Additionally, *fimH* and *aac3-II* were highly correlated with the antibiotic resistant. Meanwhile, it is important that the chosen antibiotic used in the treatment does not increase the antibiotic resistance. It is recommended to test the association between these two variables routinely, which could help elucidate the degree of UPEC pathogenicity and guide treatment decisions. Which in turn leads to less unnecessary antibiotic use.
